# The protective effects of phosphodiesterase-5 inhibitor, sildenafil on post-resuscitation cardiac dysfunction of cardiac arrest: by regulating the miR-155-5p and miR-145-5p

**DOI:** 10.1186/s13049-020-00819-5

**Published:** 2021-01-06

**Authors:** Yong He, Guoxing Wang, Chuang Li, Yuxing Wang, Qian Zhang

**Affiliations:** 1 Department of Emergency Medicine, Beijing Friendship Hospital, Capital Medical University, Beijing, China; 2 Heart Center & Beijing Key Laboratory of Hypertension, Beijing Chaoyang Hospital, Capital Medical University, Beijing, China

**Keywords:** Sildenafil, Post-resuscitation myocardial dysfunction, miR-155-5p, miR-145-5p

## Abstract

**Background:**

MiRNA-155 and miRNA-145 have been demonstrated to function as a key regulator in the development of the cardiovascular system. Recent experimental and clinical studies have indicated the cardioprotective role of sildenafil during ischemia/reperfusion (I/R) injury. This study was designed to investigate if administration of sildenafil will attenuate post-resuscitation myocardial dysfunction by regulating miRNA-155 and miR-145 expressions.

**Methods:**

Thirty-two male pigs (weighing 30 ± 2 kg) were randomly divided into 4 groups, sildenafil group (*n* = 8), sildenafil +NG-nitro-l-arginine methyl ester (L-NAME) (20 mg/kg L) group (*n* = 8), saline (SA group, *n* = 8); and sham operation group (sham group, *n* = 8). Eight minutes of untreated VF was followed by defibrillation in anesthetized, closed-chest pigs. Hemodynamic status and blood samples were obtained at 0 min, 0.5, 1, 2, 4 and 6 h after return of spontaneous circulation (ROSC), and the hearts were removed and analyzed under electron microscopy, quantitative real-time polymerase chain reaction and ultra structural analysis were performed to evaluate myocardial injury.

**Results:**

Compared with the sildenafil + L-NAME and saline groups, the sildenafil group had better outcomes in terms of hemodynamic and oxygen metabolism parameters as well as 24-h survival rate, and attenuated myocardial injury; In this study, CA pigs showed evidently increased levels of miR-155-5p and miR-145-5p, while the sildenafil treatment decreased the levels of miR-155-5p and miR-145-5p in CA pigs. In addition, the levels of eNOS was decreased in CA pigs, validating sildenafil attenuating post-resuscitation myocardial dysfunction by regulating miRNA-155 and miR-145 expressions.

**Conclusions:**

Sildenafil group had better outcomes in terms of hemodynamic and oxygen metabolism parameters as well as 24-h survival rate, inhibited the increases in the miR-155-5p and miR-145-5p levels and attenuated myocardial injury in a porcine model of CA and resuscitation.

**Supplementary Information:**

The online version contains supplementary material available at 10.1186/s13049-020-00819-5.

## Background

Morbidity and mortality from cardiac arrest (CA) remains unacceptably high, yet effective treatments for CA have proven to be elusive [1]. Global ischemia and reperfusion injury induced by cardiopulmonary resuscitation (CPR) causes so-called post-resuscitation syndrome [2]. Postresuscitation myocardiac dysfunction, an important component of the postcardiac arrest syndrome, is caused by ischemia/reperfusion (I/R) injury and includes primary manifestations such as arrhythmias, myocyte apoptosis, and contractile dysfunction [3]. Furthermore, post-resuscitation myocardial dysfunction is considered the leading cause of death within 72 h after successful CPR [4]. Therefore, studies of new medications that aim to improve post-resuscitation myocardial dysfunction are of great urgency and importance.

MicroRNAs (miRNAs/miRs) are small non-coding RNAs that are able to negatively regulate gene expression via binding to the 3′-untranslated region (UTR) of target mRNAs [5]. Previous studies have indicated that miRNAs participate in numerous cellular and molecular events, and the roles served by miRNAs in the pathogenesis of several diseases have been reported [5, 6]. MiR-155 and miR-145 have been demonstrated to function as a key regulator in the development of the cardiovascular system [7, 8]. Additionally, nitric oxide (NO) is produced by NO synthase (NOS), an enzyme present in large quantities in the endothelium, in which the expression of NOS is controlled by flow-induced shear stress [9, 10]. After I/R, endothelial dysfunction and inhibition of NOS with reduced NO availability are commonly observed, due to oxidative stress [10]. It was reported that the impairment in miRNA functions during normoxia could upregulate the expression of eNOS, thus implicating miRNAs in the general epigenetic mechanisms involving the posttranscriptional modification of eNOS expression [11]. Preliminary data suggested that miR-155 and miR-145 were shown to directly bind to NOS messenger RNA (mRNA) during normoxia [7, 8].

Sildenafil, is a selective inhibitor of the isoform 5 of the enzyme phosphodiesterase (PDE5), which is responsible for the breakdown of 39, 59-cyclic guanosine monophosphate (cGMP) in smooth muscle cells [12]. As the intracellular level of cGMP is controlled by the activity of PDE5, it is expected that pharmacological inhibition of PDE5 by sildenafil might improve cardioprotection in the myocardium [12]. Our previous animal experiments showed that sildenail improved post-resuscitation perfusion of the heart and improved cardiac function by enhancing the activation of eNOS production and acts on myocardial ischaemia-associated miRNAs [12, 13]. Additionally, it is well established that miR-155 and miR-145 are involved in the processes of I/R injury via regulating the expression of eNOS and the production of NO [7, 8]. Based on this background, the present study was designed to test the hypothesis that sildenafil attenuating post-resuscitation myocardial dysfunction by participating in the regulation of miRNA-155 and miRNA-145 expression.

## Materials and methods

### Ethics statement

This study was carried out in strict accordance with the guideline for animal care and use established by the Capital Medical University Animal Care and Use Committee. The study’s experimental protocol was approved by the Committee on the Ethics of Animal Experiments of Capital Medical University (Permit Number: 2019-D-014). Animals used in this study were handled in compliance with the Guiding Principles for the Care and Use of Animals expressed in the Declaration of Helsinki [14]. All animals were maintained in a specific pathogen-free environment in our facility, and were fed with standard chow and had free access to water. All surgery was performed under anesthesia and analgesia, and all efforts were made to minimize suffering.

### Animal preparation

Thirty-two male domestic pigs aged 11 to 13 months with an average weight of 30 ± 2 kg were used in each part of this study [15]. The strain of those pigs is mixed breed. Those animals were supplied by a single source breeder (Experimental Animal Center of Capital Medical University, Beijing, China). The piglets were randomly assigned into 4 groups, sildenafil group (*n* = 8), sildenafil +NG-nitro-l-arginine methyl ester (L-NAME) (20 mg/kg L) group (*n* = 8), saline (SA group, n = 8); and sham operation group (sham group, *n* = 8). Sildenafil was obtained from a 25-mg Viagra (Pfizer Australia) tablet that was dissolved in 50 ml saline, filtered and stored at 4 °C. In sildenafil group, this solution was given once intraperitoneally in the dose of 0.5 mg/kg 30 min prior to VF [16]. In sildenafil+ L-NAME group, sildenafil (0.5 mg/kg) and L-NAME (20 mg/kg), pretreatment was administered once intravenously at 30 min before VF [17]. The drugs were delivered in a randomized manner by the sealed envelope method, as we previously described [12]. The vehicle (0.9% NaCl) was administered in the same manner and volume. After premedication with 0.5 mg/kg intramuscular midazolam, the animal was anesthetized by ear vein injection of propofol (1.0 mg/kg) and maintained in a surgical plane of anesthesia with intravenous infusion of sodium pentobarbital (8 mg/kg/h). All animals were intubated by a cuffed 6.5-mm endotracheal tube and ventilated by a volume-controlled ventilator (Servo 900C; Siemens, Munich, Germany) using a tidal volume of 8 mL/kg and a respiratory frequency of 12 breaths/min with room air. End-tidal CO_2_ was measured by an inline infrared cacographic (CO_2_SMO plus monitor; Respirometric Inc., Murrysville). Respiratory frequency was adjusted to maintain end-tidal CO_2_ between 35 and 40 mmHg before CA was induced. Room temperature was adjusted to 26 °C, and body temperature was maintained at 37 °C under an infrared lamp, and all efforts were made to minimize suffering. Fluid losses were compensated by an infusion of 30 mL/kg acetated Ringer’s solution during the first hour of preparation, followed by a continuous infusion of 2.5% glucose-electrolytes solution 8 mL/kg/h and acetated Ringer’s solution 20 mL/kg/h. All investigators performing CPR and interpreting the outcome assessments were blinded to the medication.

An angiographic catheter was inserted from the femoral artery into the aortic arch for collecting blood samples and for measuring aortic pressure. A Swan-Ganz catheter (7 Fr; Edwards Life Sciences, Irvine, CA) was advanced from the right femoral vein and flow-directed into the pulmonary artery for measurement of right atrial pressure, mean pulmonary arterial pressure (MPAP) and cardiac output (CO). The electrocardiogram and all hemodynamic parameters were monitored with a patient monitoring system (M1165; Hewlett-Packard, Palo Alto, CA). Animals with self-adhesive defibrillation electrodes located on the chest wall. Pigs in the sham group that were not subjected to CA were used as controls. Arterial blood gas values were measured regularly using an ABL 520 Blood Gas Analyzer (Radiometer, Bronshoj, Denmark) at six time points: at baseline, 30 min, and 1, 2, 4, 6 h after ROSC. Mean aortic pressure (MAP) was monitored via the right femoral arterial catheter. The amounts of infused fluid and urine output were also monitored during the experiment. Coronary perfusion pressure (CPP) was calculated as the difference between decompression diastolic aortic and time-coincident right atrial pressure measured at the end of each minute of precordial compression. During CPR, CPP was calculated as the difference between the mean aortic and mean right atrial pressures during diastole (spontaneously beating) or decompression (CPR). CPR compression force, rate, and depth were controlled and continuously recorded during all experiments to assure that all groups received identical CPR quality.

### Experimental protocol

After establishment of vascular catheters, the animals were allowed to equilibrate for 30 min to achieve a stable resting level. Baseline measurements and arterial blood gases were obtained. Mechanical ventilation was established as described above. The temporary pacemaker conductor was inserted into the right ventricle through the right sheathing canal and connected to an electrical stimulator (GY-600A; Kaifeng Huanan Equipment Co, Ltd., Kaifeng, China) programmed in the S_1_S_2_ mode (300/200 ms), 40 V, 8:1 proportion, and 10 ms step length to provide a continuous electrical stimulus until VF [18]. VF was defined as a waveform of VF emerging on the monitor and a rapid decline in MAP toward zero. After successful induction of VF, mechanical ventilation was discontinued. Mechanical ventilation was discontinued after the onset of VF. After 8 min of untreated VF, CPR was performed. Manual chest compressions were immediately initiated at a rate of 100 compressions per minute for 2 min and ventilation conducted using a bag respirator attached to an endotracheal tube with room air. CPR was performed by the same CPR technician from our laboratory, who compressed the porcine chest to approximately one-third of the anteroposterior diameter. The quality of chest compressions was controlled by a Heart Start MRx Monitor/Defibrillator with Q-CPR (Philips Medical Systems, Best, Holland) [19]. The compression-to-ventilation ratio was 30:2. After 2 min of CPR, a single 120 J biphasic electrical shock was attempted with a Smart Biphasic defibrillator (Philips Medical Systems,Andover, MA). If the first defibrillation was unsuccessful, epinephrine (20 μg/kg) was given intravenously followed by 2 mins of CPR, and repeated every 2 min if ROSC was not achieved. The 150 J shocks were used for the second and all subsequent attempts. The study was blinded as to the medication used, and only the principal investigator, who did not take part in any resuscitation effort, knew the assignment of each animal. Furthermore, the investigators involved in data recording, data entry, and data analysis were also blinded to the allocation. If spontaneous circulation was still not achieved, CPR was continued for a further 2 min, and defibrillation was attempted once more.

ROSC was defined as 10 consecutive minutes of maintenance of systolic blood pressure at 50 mmHg. If spontaneous circulation was not restored within 30 min, we regarded the animal as dead [20]. All the animals received normal saline (10 mL/kg/h) intraoperatively to replenish fluid losses. After successful resuscitation, the animals were mechanically ventilated with 100% inspired oxygen for the first 30 min, 50% for the second 30 min and 21% thereafter. With the exception of one jugular vein sheath that was used for fluid administration, all other vascular sheaths and endotracheal tube were removed after a 6 h intensive care period. The animals were allowed to recover from anesthesia, and were then placed in observation cages and monitored for a further 18 h. After a period of 24 h, post-resuscitation measurements were completed. All catheters were removed and wounds were surgically sutured. The animals were then euthanatized with 10 mL of 10 mol/L potassium chloride intravenously following a bolus of 100 mg of propofol intravenously. Myocardial specimens were harvested and snap frozen in liquid nitrogen and stored at − 80 °C.

### Measurements

#### Hemodynamic and oxygen metabolism parameters collection

ECG was continuously monitored. The hemodynamic parameters including heart rate (HR), CO, MAP, and MPAP were measured continuously, and we recorded the values at baseline, and at 30 min, 1, 2, 4, 6 h after ROSC. At the end of each time point, 4 °C saline was injected into the right atrium through the Swan-Ganz catheter to determine CO by the transpulmonary thermo dilution method as described previously [12]. MAP was determined by the electronic integration of the aortic blood pressure waveform. The amounts of infused fluid and urine output were also monitored during the experiment. Serum lactate level, and arterial blood gas values of which temperatures were corrected to 37 °C were measured regularly using an ABL 520 Blood Gas Analyzer (Radiometer, Bronshoj, Denmark). CPP was calculated as the difference between decompression diastolic aortic and time-coincident right atrial pressure measured at the end of each minute of precordial compression. CPP during VF was defined as the difference between the mean aortic and mean right atrial pressures. Oxygen metabolism parameters, including oxygen delivery (DO_2_) and oxygen consumption (VO_2_), were calculated.

#### Survival

The survival rate was determined based on the animals that survived the experimental protocol starting at ROSC until 24 h after ROSC. Animals that died during surgical recovery were excluded.

#### Micro-RNA isolation and expression

Total RNA samples were extracted using Trizol (Invitrogen, USA) from cultural myocytes. MiR-155-5p, miR-145-5p and eNOS level were quantified by the mirVana qRT-PCR (quantitative real-time PCR) miRNA Detection Kit (Ambion, USA) in conjunction with real-time PCR with SYBR Green I (Applied Biosystems, USA) [21]. The following primers were used for PCR detection: miR-155-5p [5′-GCGCGTTAATGCTAATTGTGA-3′(forward); 5′-AGTGCAGGGTCCGAGGTATT-3′ (reverse)]; miR-145-5p [5′- CGGTCCAGTTTTCCCAGGAA − 3′ (forward); 5′ AGTGCAGGGTCCGAGGTATT − 3′ (reverse)], U6 was used as an internal control. eNOS [5′-TCC CAG ACC CCA TAA CAA CAG-3′ (sense) and 5′-TGA GGG TGC AGCGAA CTT TA-3′ (antisense)]. The relative expression of miR-155-5p, miR-145-5p and eNOS mRNA was calculated using the 2 − ΔΔCt method. All samples were run in triplicate from three independent experiments.

#### Ultra structural analysis

The remaining tissue was preserved in 10% formaldehyde and 4% paraformaldehyde to observe pathologic and ultra structural changes of the myocardium under transmission electron microscope (TEM) (H-7650; Hitachi, Tokyo, Japan). The pathologic data were assessed by reviewers blinded to the experimental groups.

#### Statistical analyses

Continuous variables were presented as mean ± standard deviation (SD) when data were normally distributed or as a median (25th, 75th percentiles) when data were not normally distributed. Student *t* test was used for comparisons between every two groups. Differences at different time points were compared with repeated-measures analysis of variance (ANOVA) with Bonferroni correction for post hoc comparison. The Kruskal-Wallis test was used to compare continuous variables in multiple groups. For these comparisons, the Bonferroni correction was applied to control for the multiple testing. Survival analysis was performed using the method of Kaplan and Meier, and comparisons between groups were made using the log-rank test. A value of *p* < 0.05 was considered as statistically significant. All analyses were conducted using the SPSS 17.0 software (SPSS Inc., Chicago III) and GraphPad PRISM version 6 (GraphPad Software Inc., San Diego, CA).

## Results

### Baseline status

Baseline hemodynamic measurements and oxygen metabolism measurements are shown in (Table 1). None of the variables (body weight, HR, MAP, MPAP, CO, lactate concentration, DO_2_, VO_2_ and extraction of oxygen (ERO_2_) differed significantly among the four groups (*p* > 0.05).

**Table 1:** Baseline characteristics

	SHAM group (***n*** = 8)	SA group (***n*** = 8)	Sildenafil group (***n*** = 8)	Sildenafil + L-NANE group (***n*** = 8)
Weight (kg)	29.13 ± 2.16	30.63 ± 0.92	30.38 ± 0.92	30.44 ± 0.89
HR	99.00 ± 7.44	101.38 ± 8.30	100.50 ± 10.04	101.52 ± 9.84
MAP, mmHg	103.12 ± 5.19	101.88 ± 5.22	99.00 ± 5.81	102.41 ± 5.21
MPAP, mmHg	23.42 ± 4.32	24.13 ± 5.24	24.34 ± 4.56	23.72 ± 4.68
CO, L/min	2.86 ± 0.22	2.99 ± 0.20	2.99 ± 0.19	2.89 ± 0.22
DO_2_, ml/min	444 ± 39	445 ± 34	450 ± 28	439 ± 31
VO_2_, ml/min	112 ± 12	112 ± 9	115 ± 12	113 ± 10
ERO_2_, %	24.17 ± 2.34	25.44 ± 2.70	25.46 ± 1.49	25.36 ± 1.76
Lac, mmol/L	1.21 ± 0.49	1.31 ± 0.72	1.23 ± 0.401	1.24 ± 0.42

Values are mean ± SD or number (n)

*SHAM* sham, *SA* saline, *HR* heart rate, *CO* cardiac output; *MAP* mean aortic pressure, *MPAP* mean pulmonary arterial pressure, *DO*_*2*_ oxygen delivery, *VO*_*2*_ oxygen consumption, *ERO*_*2*_ extraction of oxygen, *Lac* lactate

### Resuscitation outcomes and survival

**Fig. 1:**
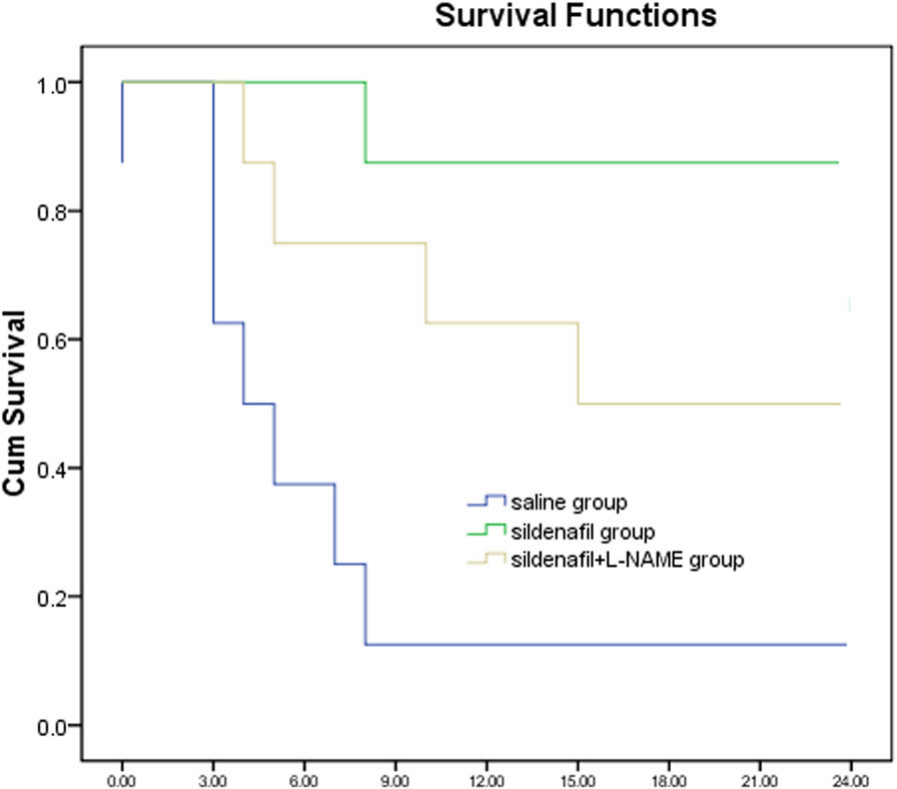
Kaplan-Meier survival curve. We found a significant difference in survival between the sildenafil group, sildenafil + L-NAME group and the saline group (*P* < 0.001 by logrank test)

Resuscitation outcomes are shown in Table 2. None of the 24 animals restored spontaneous circulation after initial defibrillation attempts. By comparison, the cumulative defibrillation energy was significantly lower in sildenafil group than in sildenafil + L-NAME group and SA group (*p* < 0.05). ROSC was achieved in eight piglets in the both sildenafil group and in the sildenafil + L-NAME group, and in seven of eight piglets in the SA group. In the SA group, six piglets died at 14mins, 70 mins, 196 mins, 256 mins, 322 mins and 416 mins after ROSC. In the sildenafil + L-NAME group, four piglets died at 10 mins, 75 mins, 180 mins and 210 mins after ROSC. While, in the sildenafil group, only one piglet died at 48 min after ROSC. A significant difference in survival to the end of the 24-h experiment between sildenafil, sildenafil + L-NAME and SA groups were demonstrated using the Kaplan-Meier survival curve and the log-rank test (*p* < 0.05) (Fig. 1).

**Table 2:** Cardiopulmonary resuscitation outcomes

	SA group (***n*** = 8)	Sildenafil group (***n*** = 8)	Sildenafil + L-NAME group (***n*** = 8)
Number of defibrillatory shocks	4.31 ± 1.62	2.91 ± 0.83	3.72 ± 0.71
Cumulative defibrillation energy (J)	145.01 ± 33.41	95.02 ± 33.41*^#^	115.23 ± 23.14*
Duration of CPR before ROSC (min)	6.12 ± 2.21	4.71 ± 1.32	5.43 ± 1.58
6-h survival	3	7*	6*
24-h survival	1	7*^#^	4*

Values are mean ± SD or number (n)

*SA* saline, *ROSC* restoration of spontaneous circulation, *CPR* cardiopulmonary resuscitation

*p < 0.05 vs. SA group, ^#^*p* < 0.05 vs. Sildenafil+L-NAME group

### Hemodynamics and left ventricular function

Indeed, HR, MAP and MPAP were significantly increased during the first 4 h after ROSC compared to baseline in the three resuscitation groups (*p* < 0.01 vs. Baseline, Table 2). However, sildenafil pretreatment inhibited this increase of HR, MAP and MPAP at four time-points (0 min, 30 min, 2 h and 4 h after ROSC), while the values of CPP and CO were decreased during the initial 6 h after ROSC when compared to baseline values (*p* < 0.01, respectively) and the CO and CPP values were significantly higher in the sildenafil group than those in the sildenafil + L-NAME group at 1, 2 h and 4 h after ROSC (*p* = 0.03, *p* = 0.02, *p* = 0.04, respectively (Table 3).

**Table 3:** Haemodynamics during cardiopulmonary resuscitation and ROSC

Group	Parameter	Baseline	ROSC 0 min	ROSC 30 min	ROSC 1 h	ROSC 2 h	ROSC 4 h	ROSC 6 h
**SA group**	**HR**	101.3 ± 8.30	132.2 ± 11.3^**^	129.4 ± 10.4^**^	120.3 ± 8.9^**^	118.9 ± 9.3^**^	113.5 ± 17.4^*^	101.4 ± 10.4
	**CO**	2.99 ± 0.20	0.91 ± 0.07^**^	0.88 ± 0.06^**^	0.89 ± 0.06^**^	0.91 ± 0.05^**^	1.04 ± 0.05^**^	1.15 ± 0.04^**^
	**MAP**	91.88 ± 3.22	125.2 ± 7.3^**^	122.5 ± 6.4^**^	115.4 ± 6.1^**^	109.8 ± 5.7^**^	107.6 ± 5.1^**^	101.5 ± 4.8^*^
	**CPP**	44.2 ± 4.12	22.3 ± 2.5^**^	23.4 ± 2.6^**^	25.6 ± 2.7^**^	29.7 ± 3.6^**^	32.5 ± 3.8^*^	33.8 ± 3.7^*^
	**MPAP**	24.1 ± 2.2	49.7 ± 6.3^**^	47.3 ± 5.7^**^	43.8 ± 4.7^**^	39.7 ± 4.2^**^	35.8 ± 3.7^*^	35.1 ± 3.1^*^
**Sildenafil group**	**HR**	102.1 ± 7.30	123.2 ± 9.3^**Δ#^	118.4 ± 8.4^**Δ#^	115.3 ± 7.9^**^	110.9 ± 6.3^*Δ#^	107.5 ± 5.4^Δ#^	105.4 ± 4.4
	**CO**	2.99 ± 0.20	0.99 ± 0.06^**^	1.12 ± 0.03^**^	1.23 ± 0.04^**Δ#^	1.34 ± 0.08^**Δ#^	1.42 ± 0.07^**Δ#^	1.36 ± 0.08^**^
	**MAP**	89.88 ± 3.22	117.2 ± 7.1^**Δ#^	115.5 ± 5.9^**Δ#^	111.8 ± 5.6^**^	103.6 ± 4.7^**Δ#^	99.8 ± 4.3^*Δ#^	94.5 ± 3.4
	**CPP**	43.1 ± 3.34	27.6 ± 2.8^**Δ^	28.2 ± 2.9^**Δ^	32.4 ± 3.1^**^	35.4 ± 3.6^*Δ#^	36.5 ± 3.9^Δ#^	39.4 ± 4.3^Δ^
	**MPAP**	24.6 ± 2.7	40.3 ± 5.6^**Δ#^	38.4 ± 4.1^**Δ#^	37.8 ± 4.1^**Δ#^	33.6 ± 3.5^*Δ^	29.8 ± 3.1^Δ^	28.7 ± 2.2^Δ^
**Sildenafil + L-NANE group**	**HR**	103.2 ± 7.22	128.4 ± 9.1^**^	125.6 ± 7.3^*Δ^	118.5 ± 7.2^*^	115.9 ± 6.6^*Δ^	112.6 ± 5.7^Δ^	104.7 ± 6.4
	**CO**	2.99 ± 0.20	0.98 ± 0.07^**^	0.87 ± 0.05^**^	0.92 ± 0.06^**^	0.98 ± 0.04^**^	1.17 ± 0.05^**^	1.21 ± 0.06^**^
	**MAP**	88.18 ± 3.22	121.2 ± 6.1^**Δ^	120.6 ± 7.4^**^	117.4 ± 6.4^**^	113.8 ± 6.7^**^	110.7 ± 4.6^**^	99.7 ± 4.6^*^
	**CPP**	44.2 ± 3.52	24.8 ± 2.7^**Δ^	25.7 ± 2.8^**^	27.7 ± 2.6^**^	30.6 ± 3.2^**^	32.7 ± 3.6^*^	35.7 ± 3.4^*^
	**MPAP**	24.6 ± 2.7	45.2 ± 5.4^**Δ^	46.2 ± 5.3^**^	44.7 ± 4.3^**^	38.4 ± 4.7^**^	36.9 ± 3.5^*^	34.3 ± 3.6^*^

Values are mean ± SD (n)

*p < 0.05 vs. baseline, **p < 0.01 vs. baseline, Δ *P* < 0.05 vs. SA group. # *p* < 0.05 vs. Sildenafil+L-NAME group. (Student t test was used for comparisons between every two groups. Partial pressures are given in mmHg. HCO3: bicarbonate in mM; Differences at different time points were compared with repeated-measures analysis of variance (ANOVA) with Bon-ferroni correction for post hoc comparison between multiple experimental groups)

### Arterial blood gases

There were no significant differences in blood gas values at baseline among three resuscitation groups. The serum levels of PH, HCO_3_^−^, PaO_2_, PaCO_2_ and lactate were significantly worse than those at baseline in three resuscitation groups. PH, HCO_3_^−^, PaCO_2_ and lactate were significantly lower in the sildenafil group than those in the sildenafil + L-NAME group at 0 min, 30 min and 1 h after ROSC (*p* = 0.04, *p* = 0.02, *p* = 0.02, *p* = 0.03 respectively) and the PaO2 was higher in the sildenafil group than those in the sildenafil + L-NAME group at 0 min, 30 min, 1 h and 2 h after ROSC (*p* = 0.03, *p* = 0.04, *p* = 0.02, *p* = 0.03, respectively, Table 4).

**Table 4:** Arterial blood gasses

Group	Parameter	Baseline	ROSC 0 min	ROSC 30 min	ROSC 1 h	ROSC 2 h	ROSC 4 h	ROSC 6 h
**SA group**	**PH**	7.41 ± 0.11	7.14 ± 0.05^**^	7.18 ± 0.07^**^	7.20 ± 0.06^**^	7.27 ± 0.09^*^	7.30 ± 0.08^*^	7.32 ± 0.09^*^
	**PaO**_**2**_ **(mm Hg)**	89.1 ± 9.3	44.5 ± 4.7^**^	55.4 ± 5.1^**^	60.1 ± 6.2^**^	70.2 ± 7.1^**^	79.5 ± 7.7^*^	80.7 ± 7.5^*^
	**PaCO**_**2**_ **(mm Hg)**	39.3 ± 3.2	48.3 ± 5.1^**^	46.7 ± 5.4^**^	45.4 ± 4.1^**^	43.8 ± 4.7^*^	41.6 ± 4.2	41.5 ± 4.8
	**HCO**_**3**_**(mmol/L)**	27.2 ± 4.7	19.3 ± 1.2^**^	20.4 ± 1.6^**^	21.6 ± 2.7^**^	22.7 ± 2.6^**^	24.5 ± 3.1^*^	25.2 ± 3.7
	**LAC (mmol/L)**	2.6 ± 0.5	11.2 ± 3.3^**^	9.3 ± 2.7^**^	8.8 ± 1.9^**^	6.9 ± 2.2^**^	5.8 ± 1.3^*^	4.7 ± 1.1^*^
**Sildenafil group**	**PH**	7.42 ± 0.13	7.27 ± 0.07^**Δ#^	7.29 ± 0.04^**Δ#^	7.30 ± 0.07^*Δ#^	7.32 ± 0.05^*^	7.35 ± 0.04	7.38 ± 0.11
	**PaO**_**2**_ **(mm Hg)**	90.2 ± 8.6	52.7 ± 5.3^**Δ#^	63.5 ± 6.2^**Δ#^	70.3 ± 7.1^**Δ#^	79.3 ± 7.7^*Δ#^	83.5 ± 8.1^*^	84.9 ± 8.2
	**PaCO**_**2**_ **(mm Hg)**	39.5 ± 3.4	44.7 ± 4.3^**Δ#^	43.2 ± 4.4^*Δ#^	42.4 ± 4.1^*Δ#^	41.3 ± 4.2	40.5 ± 4.2	41.1 ± 3.8
	**HCO**_**3**_ **(mmol/L)**	27.6 ± 4.1	22.6 ± 2.7^**Δ#^	23.7 ± 2.6^**Δ#^	24.6 ± 2.7^*Δ#^	25.3 ± 2.6	25.7 ± 3.1	26.3 ± 3.8
	**LAC (mmol/L)**	2.7 ± 0.4	8.5 ± 2.4^**Δ#^	8.1 ± 2.1^**Δ#^	7.4 ± 1.6^**Δ#^	5.3 ± 1.3^**Δ^	4.2 ± 1.2^*^	3.6 ± 0.7
**Sildenafil + LNANE group**	**PH**	7.41 ± 0.13	7.19 ± 0.06^**Δ^	7.21 ± 0.06^**Δ^	7.23 ± 0.04^**Δ^	7.29 ± 0.09^**^	7.31 ± 0.07^**^	7.34 ± 0.07^*^
	**PaO**_**2**_ **(mm Hg)**	90.1 ± 9.1	46.4 ± 3.7^**^	58.5 ± 4.3^**^	63.1 ± 6.2^**^	72.2 ± 7.1^**^	80.5 ± 7.7^*^	80.9 ± 7.3^*^
	**PaCO**_**2**_ **(mm Hg)**	39.4 ± 3.3	47.8 ± 4.4^**^	46.3 ± 4.3^**^	44.6 ± 4.5^**^	43.2 ± 4.1^*^	42.3 ± 3.3	41.3 ± 4.2
	**HCO**_**3**_ **(mmol/L)**	27.4 ± 4.3	20.5 ± 2.4^**^	21.7 ± 2.5^**^	21.6 ± 2.7^**^	22.7 ± 2.6^**^	23.8 ± 3.2^*^	25.4 ± 3.6
	**LAC** **(mmol/L)**	2.6 ± 0.4	10.8 ± 4.5^**^	9.8 ± 3.5^**^	8.8 ± 1.9^**^	6.9 ± 2.2^**^	5.8 ± 1.3^*^	4.1 ± 2.3

Values are mean ± SD (n)

^*^p < 0.05 vs. baseline, ^**^p < 0.01 vs. baseline, ^Δ^ *P* < 0.05 vs. SA.^#^ *P* < 0.05 vs. Sildenafil+L-NAME group. (Student *t* test was used for comparisons between every two groups. Partial pressures are given in mmHg. HCO_3_: bicarbonate in mM; Differences at different time points were compared with repeated-measures analysis of variance (ANOVA) with Bon-ferroni correction for post hoc comparison between multiple experimental groups)

### Effect of sildenafil on expression of miR-155-5p and miR-145-5p

**Fig. 2:**
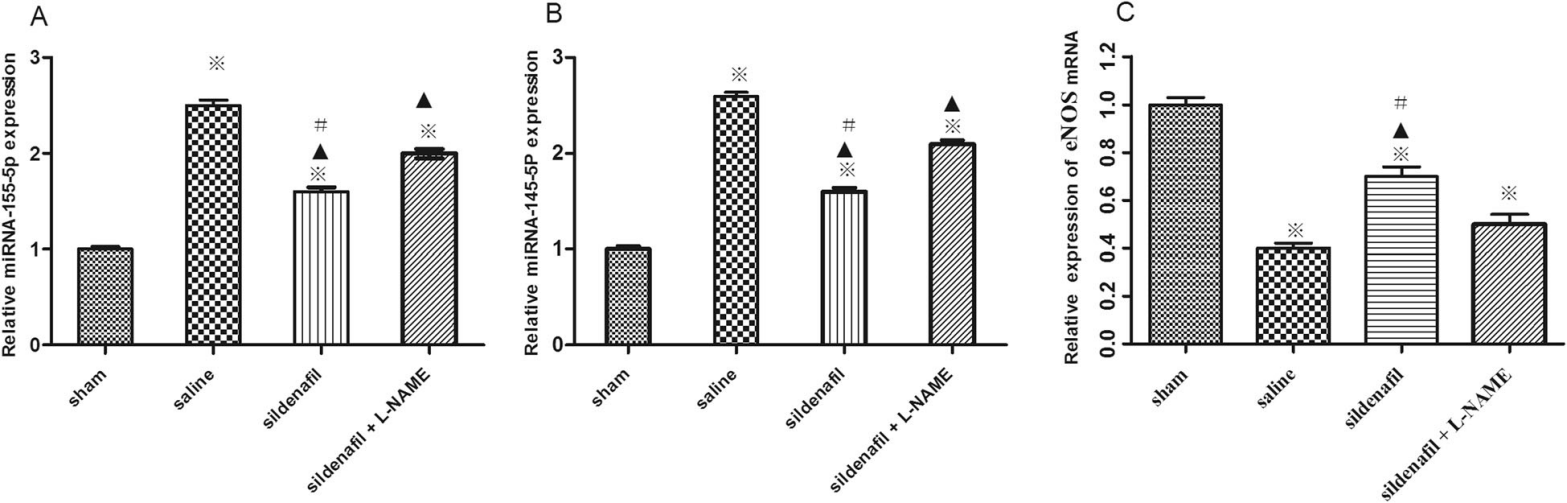
Real-time PCR analysis of relative microRNA-155-5p (**a**), microRNA-145-5p (**b**) and eNOS (**c**) expression. The value represent mean ± SD. *p < 0.05 vs.sham, ^▲^ *p* < 0.05 vs.saline, #*p* < 0.05 vs. sildenafil + L-NAME group

Real-time PCR analysis revealed that following CA and post-resuscitation treatment pigs showed evidently increased levels of miR-155-5p (Fig. 2a) and miR-145-5p (Fig. 2b) compared with sham operated pigs, whereas that there were the significantly down-regulated expression of miR-155 and miR-145 following sildenafil treatment compared with the sildenafil + L-NAME group and SA group ((both *p* < 0.01). Similarly, compared with that in the sham group, the relative expression of eNOS mRNA (Fig. 2c) were suppressed in the following CA and post-resuscitation groups, while the expression of eNOS mRNA was recovered in the sildenafil treatment group. The results suggested that sildenafil may serve a protective role in post-resuscitation myocardial dysfunction by participating in the regulation of miRNA-155 and miRNA-145 expression.

### Ultra structural changes in cardiomyocytes

**Fig. 3:**
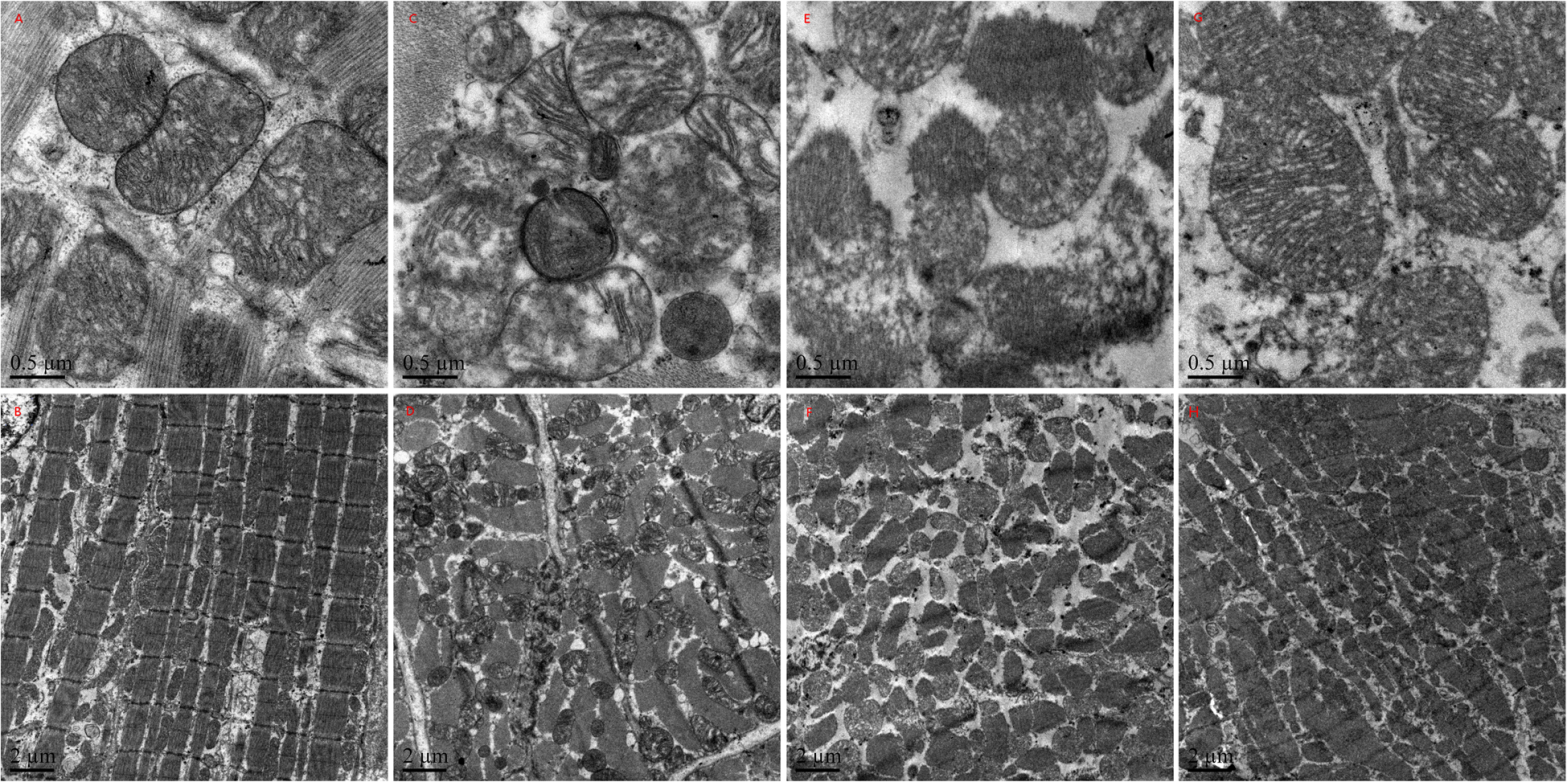
Cytoplasmic ultrastructure of the myocardium under an electron microscope: **a, b**: normal mitochondria structures were displayed, Z-line, M-line and intercalated disk is clear in the sham group. **c, d**: The myocardial fiber and intercalated disk were obviously disordered, broken, even dissolved in the SA group 24 h after ROSC. **e, f**: Animals treated with sildenafil+ L-NAME exhibited lighter intracellular damage in the myocardium at 24 h after ROSC than the SA group. **g, h**: Animals treated with sildenafil exhibited little intracellular damage in the myocardium at 24 h after ROSC

Under TEM, normal mitochondria structures were displayed in the sham group (Fig. 3a, b). The myocardial fiber and intercalated disk were obviously disordered, broken, even dissolved in the SA group 24 h after ROSC; most of the mitochondria were severely broken, even exhibiting vascular with vague, arranged irregularly, or disrupted cristae (Fig. 3c, d). However, all of them were improved faster in the sildenafil than in the sildenafil + L-NAME group and in the SA group (Fig. 3e-h). Additionally, myocardial damage were further significantly alleviated in the sildenafil group when compared with the sildenafil + L-NAME group, animals treated with sildenafil exhibited little intracellular damage in the myocardium: partial nuclear chromatin condensation, reduced crest fracture and moderate edema occurred in the mitochondria and sarcoplasmic reticula (Fig. 3g, h).

## Discussion

The major findings of this study were as follows: (1) Compared with the sildenafil + L-NAME and saline groups, the sildenafil group had better outcomes in terms of hemodynamic and oxygen metabolism parameters as well as 24-h survival rate, and attenuated myocardial injury in a porcine model of CA and resuscitation. (2) In this study, CA pigs showed evidently increased levels of miR-155-5p and miR-145-5p, while the sildenafil treatment decreased the levels of miR-155-5p and miR-145-5p in CA pigs. In addition, the levels of eNOS was decreased in CA pigs, validating sildenafil attenuating post-resuscitation myocardial dysfunction by regulating miRNA-155 and miR-145 expressions.

The severity of postresuscitation myocardial dysfunction is the major cause of early death, and no effective treatment method has been established [3]. Actively protective treatment to myocardial function is urgent to improve postresuscitation outcomes. Therefore, the study of postresuscitation myocardial dysfunction and the identification of drugs to treat postresuscitation myocardial dysfunction have attracted much attention. Our previous research demonstrated that sildenafil improved post-resuscitation perfusion of the heart, and reduced cardiac myocyte apoptosis and improved cardiac function [12]. Additionally, both animal and clinical studies have consistently demonstrated that the severity of postresuscitation myocardial dysfunction is closely associated with the duration of ischemia, number of electrical defibrillations [22, 23]. In the present study, the administration of sildenafil significantly alleviated the severity of postresuscitation myocardial dysfunction and myocardial tissue injuries, as reflected by improvements in HR, MAP, MPAP and PaO2 values, and myocardial tissue morphological injuries. The results of sildenafil administration on postresuscitation myocardial dysfunction and the 24 h survival rate in a pig CA model suggest that this drug may have further clinical applications and research value for CA patients.

NO is an endothelium-derived vasoactive factor produced by NOS, which plays important roles in modulating coronary vascular tone and tissue perfusion. After I/R, endothelial dysfunction and inhibition of NOS with reduced NO availability are commonly observed, due to oxidative stress [10]. Evidence indicates that NO through the NO-NOS/GC/cGMP (nitric oxide/nitric oxide synthase-guanyly cyclase-cyclic guanosine mono phosphate) pathway has an important function in the development of myocardial dysfunction with I/R injury [24]. The protective mechanisms of sildenafil appear to be due to activation of the NO/cGMP signaling pathway via the inhibition of PDE5 activity and coordinated induction of NOS isoform expression [12, 13]. Subsequently, cGMP may activate protein kinase G that in turn opens the KATP channel, resulting in the cardioprotective effects as reported earlier [12]. Our results confirmed that that sildenafil playing a critical role in post-resuscitation myocardial dysfunction by regulating iNOS/eNOS and miRNAs expression. We also demonstrated that all animals had different degrees of myocardial injury after CA and resuscitation. Our study showed that the impairment of myocardial dysfunction and injury could be reversed by sildenafil.

Another focus of this study is the molecular mechanism underlying the myocardial protection provided by sildenafil in a pig CA model. Certain target genes of miR-155 and miR-145 have been identified and demonstrated to serve important regulatory roles in a number of cellular processes in previous studies. Previous studies have also shown that miR-155 and miR-145 participated in the regulation of a wide range of cellular functions, such as macrophage derived foam cell formation, viral infection, inflammation, immunologic response, and hematopoiesis [25, 26]. Cytokines, such as tumor necrosis factor-α, were shown to increase the expression of miR-155 in human umbilical vein endothelial cells [27]. A recent study has shown that miR-155 reduced eNOS expression by decreasing the stability of eNOS mRNA [7]. In fact, upon cytokine activation, the inhibition of miR-155 expression prevented the reduction in NO production and eNOS expression [7]. Santovito et al. examined the expression of miR-145 in the atherosclerotic plaques of patients with and without essential hypertension, and identified that miR-145 was overexpressed in patients with hypertension [28]. Wang et al. observed that miR-145 negatively regulates the production of NO through targeting SLC7A1 [29]. These results suggest that miR-155 and miR-145 serve key roles in regulating cell function and blood pressure. Our research confirmed that CA and resuscitation significantly increased the expression of miR-155-5p and miR-145-5p in myocardial tissue, and the administration of sildenafil significantly decreased the expression of miR-155 and miR-145. These results suggest that the protective mechanism of sildenafil may be related to regulate miRNAs expression.

To interpret the results of our experimental study, it is necessary to take several limitations into consideration. First, this study was performed in animals without any underlying diseases related to cardiac arrest [30]. Second, we did not examine different doses of sildenafil in this study. Third, this experiment did not serially measure myocardial tissue injury biomarkers from 30 min to 24 h after ROSC. Forth, based on previous animal research and pharmacokinetics of sildenafil, the drugs were administered as a pre-treatment 30 min before VF which cannot reflect its clinical applicability truly. Fifth, the long-term treatment outcomes and prognosis of sildenafil therapy after ROSC require further studies. Finally, we plan to investigate the other possible signals mediating the effect of sildenafil on post-resuscitation myocardial dysfunction.

## Conclusions

The present study demonstrated that sildenafil could improve the outcomes in terms of hemodynamic and oxygen metabolism parameters as well as 24-h survival rate, inhibite the increases in the miR-155-5p and miR-145-5p levels and attenuate myocardial injury in a porcine model of CA and resuscitation. Sildenafil has the potential to improve post-resuscitation myocardial dysfunction in patients with clinical conditions induced by CPR.

## Supplementary Information

**Additional file 1:** Animal Research Reporting In Vivo Experiments.

## Data Availability

All data generated or analyzed during this study are included in this published article.

## Abbreviations

CA: Cardiac arrest
cGMP: Cyclic guanosine monophosphate
CO: Cardiac output
CPP: Coronary perfusion pressure
CPR: Cardiopulmonary resuscitation
EF: Ejection fraction
HR: Heart rate
I/R: Ischemia/reperfusion
MAP: Mean aortic pressure
MPAP: Mean pulmonary arterial pressure
NO: Nitrogen oxide
PDE-5: Isoform 5 of the enzyme phosphodiesterase
ROSC: Return of spontaneous circulation
SA: Saline
SHAM: Sham
VO_2_: Oxygen consumption
