# Supplementary material for: The protective effects of phosphodiesterase-5 inhibitor, sildenafil on post-resuscitation cardiac dysfunction of cardiac arrest: by regulating the miR-155-5p and miR-145-5p
Source: Scand J Trauma Resusc Emerg Med. 2021 Jan 6;29:2. doi: 10.1186/s13049-020-00819-5 (PMC7787123; doi:10.1186/s13049-020-00819-5)
Supplement: Supplementary file 1 — Additional file 1: Animal Research Reporting In Vivo Experiments. [file 13049_2020_819_MOESM1_ESM.doc]

**Additional file 1: The ARRIVE Guidelines**

**Animal Research: Reporting *In Vivo* Experiments**

|  | **Item** | **Recommendation** |
| --- | --- | --- |
| **TITLE** | **1** | **The protective effects of phosphodiesterase-5 inhibitor, sildenafil on post-resuscitation cardiac dysfunction of cardiac arrest: by regulating the miR-155-5p and miR-145-5p** |
| **ABSTRACT** | **2** | **Introduction** MiRNA-155 and miRNA-145 have been demonstrated to function as a key regulator in the development of the cardiovascular system. Recent experimental and clinical studies have indicated the cardioprotective role of sildenafil during ischemia/reperfusion (I/R) injury. This study was designed to investigate if administration of sildenafil will attenuate post-resuscitation myocardial dysfunction by regulating miRNA-155 and miR-145 expressions.  **Methods** Thirty-two male pigs (weighing 30 ± 2 kg) were randomly divided into 4 groups, sildenafil group (n= 8), sildenafil +NG-nitro-l-arginine methyl ester (L-NAME) (20mg/kg L) group (n=8), saline (SA group, n=8); and sham operation group (sham group, n=8). Eight minutes of untreated VF was followed by defibrillation in anesthetized, closed-chest pigs. Hemodynamic status and blood samples were obtained at 0 min, 0.5, 1, 2, 4 and 6h after return of spontaneous circulation (ROSC), and the hearts were removed and analyzed under electron microscopy, quantitative real-time polymerase chain reaction and ultra structural analysis were performed to evaluate myocardial injury.  **Results** Compared with the sildenafil + L-NAME and saline groups, the sildenafil group had better outcomes in terms of hemodynamic and oxygen metabolism parameters as well as 24-hour survival rate, and attenuated myocardial injury; In this study, CA pigs showed evidently increased levels of miR-155-5p and miR-145-5p, while the sildenafil treatment decreased the levels of miR-155-5p and miR-145-5p in CA pigs. In addition, the levels of eNOS was decreased in CA pigs, validating sildenafil attenuating post-resuscitation myocardial dysfunction by regulating miRNA-155 and miR-145 expressions.  **Conclusions** sildenafil group had better outcomes in terms of hemodynamic and oxygen metabolism parameters as well as 24-hour survival rate, inhibited the increases in the miR-155-5p and miR-145-5p levels and attenuated myocardial injury in a porcine model of CA and resuscitation. |
| **INTRODUCTION** |  |  |
| - **Background** | **3** | Morbidity and mortality from cardiac arrest (CA) remains unacceptably high, yet effective treatments for CA have proven to be elusive. Global ischemia and reperfusion injury induced by cardiopulmonary resuscitation (CPR) causes so-called post-resuscitation syndrome. Postresuscitation myocardiac dysfunction, an important component of the postcardiac arrest syndrome, is caused by ischemia/reperfusion (I/R) injury and includes primary manifestations such as arrhythmias, myocyte apoptosis, and contractile dysfunction. Furthermore, post-resuscitation myocardial dysfunction is considered the leading cause of death within 72 h after successful CPR. Therefore, studies of new medications that aim to improve post-resuscitation myocardial dysfunction are of great urgency and importance.  MicroRNAs (miRNAs/miRs) are small non-coding RNAs that are able to negatively regulate gene expression via binding to the 3'-untranslated region (UTR) of target mRNAs. Previous studies have indicated that miRNAs participate in numerous cellular and molecular events, and the roles served by miRNAs in the pathogenesis of several diseases have been reported. MiR-155 and miR-145 have been demonstrated to function as a key regulator in the development of the cardiovascular system. Additionally, nitric oxide (NO) is produced by NO synthase (NOS), an enzyme present in large quantities in the endothelium, in which the expression of NOS is controlled by flow-induced shear stress. After I/R, endothelial dysfunction and inhibition of NOS with reduced NO availability are commonly observed, due to oxidative stress. It was reported that the impairment in miRNA functions during normoxia could upregulate the expression of eNOS, thus implicating miRNAs in the general epigenetic mechanisms involving the posttranscriptional modification of eNOS expression. Preliminary data suggested that miR-155 and miR-145 were shown to directly bind to NOS messenger RNA (mRNA) during normoxia.  Sildenafil, is a selective inhibitor of the isoform 5 of the enzyme phosphodiesterase (PDE5), which is responsible for the breakdown of 39, 59-cyclic guanosine monophosphate (cGMP) in smooth muscle cells. As the intracellular level of cGMP is controlled by the activity of PDE5, it is expected that pharmacological inhibition of PDE5 by sildenafil might improve cardioprotection in the myocardium. Our previous animal experiments showed that sildenail improved post-resuscitation perfusion of the heart and improved cardiac function by enhancing the activation of eNOS production and acts on myocardial ischaemia-associated miRNAs. Additionally, it is well established that miR-155 and miR-145 are involved in the processes of I/R injury via regulating the expression of eNOS and the production of NO. |
| - **Objectives** | **4** | Based on this background, the present study was designed to test the hypothesis that sildenafil attenuating post-resuscitation myocardial dysfunction by participating in the regulation of miRNA-155 and miRNA-145 expression. |
| **METHODS** |  |  |
| - **Ethical statement** | **5** | This study was approved by the Institutional Animal Care and Use Committee of the Capital Medical University and performed at the Beijing Chao-Yang Hospital Affiliated to the Capital Medical University. |
| - **Study design** | **6** | **RCT** |
| - **Experimental procedures** | **7** | Thirty-two male domestic pigs aged 11 to 13 months with an average weight of 30 ± 2 kg were used in each part of this study [15]. The strain of those pigs is mixed breed. Those animals were supplied by a single source breeder (Experimental Animal Center of Capital Medical University, Beijing, China).The piglets were randomly assigned into 4 groups, sildenafil group (n= 8), sildenafil +NG-nitro-l-arginine methyl ester (L-NAME) (20mg/kg L) group (n=8), saline (SA group, n=8); and sham operation group (sham group, n= 8). Sildenafil was obtained from a 25-mg Viagra (Pfizer Australia) tablet that was dissolved in 50 ml saline, filtered and stored at 4℃. In sildenafil group, this solution was given once intraperitoneally in the dose of 0.5 mg/kg 30 min prior to VF. In sildenafil+ L-NAME group, sildenafil (0.5 mg/kg) and L-NAME (20mg/kg), pretreatment was administered once intravenously at 30 min before VF . |
| - **Experimental animals** | **8** | **Pigs** |
| - **Housing and husbandry** | **9** | All protocols strictly conformed to the National Research Council’s 1996 Guide for the Care and Use of Laboratory Animals. |
| - **Sample size** | **10** | **32** |
| - **Allocating animals to experimental groups** | **11** | **Yes** |
| - **Experimental outcomes** | **12** | None of the 24 animals restored spontaneous circulation after initial defibrillation attempts. By comparison, the cumulative defibrillation energy was significantly lower in sildenafil group than in sildenafil + L-NAME group and SA group (p< 0.05). ROSC was achieved in eight piglets in the both sildenafil group and in the sildenafil + L-NAME group, and in seven of eight piglets in the SA group. In the SA group, six piglets died at 14mins, 70 mins, 196 mins, 256 mins, 322 mins and 416 mins after ROSC. In the sildenafil + L-NAME group, four piglets died at 10 mins, 75 mins, 180 mins and 210 mins after ROSC. While, in the sildenafil group, only one piglet died at 48 minute after ROSC. |
| - **Statistical methods** | **13** | Continuous variables were presented as mean ± standard deviation (SD) when data were normally distributed or as a median (25th, 75th percentiles) when data were not normally distributed. Student *t* test was used for comparisons between every two groups. Differences at different time points were compared with repeated-measures analysis of variance (ANOVA) with Bonferroni correction for post hoc comparison. The Kruskal-Wallis test was used to compare continuous variables in multiple groups. For these comparisons, the Bonferroni correction was applied to control for the multiple testing. Survival analysis was performed using the method of Kaplan and Meier, and comparisons between groups were made using the log-rank test. A value of p < 0.05 was considered as statistically significant. All analyses were conducted using the SPSS 17.0 software (SPSS Inc, Chicago III) and GraphPad PRISM version 6 (GraphPad Software Inc.,San Diego,CA). |
| **RESULTS** |  |  |
| - **Baseline data** | **14** | Baseline hemodynamic measurements and oxygen metabolism measurements are shown in (Table 1). None of the variables (body weight, HR, MAP, MPAP, CO, lactate concentration, DO_2_, VO_2_ and extraction of oxygen (ERO_2_) differed significantly among the four groups (p>0.05). |
| - **Numbers analyzed** | **15** | **32** |
| - **Outcomes and estimation** | **16** | None of the 24 animals restored spontaneous circulation after initial defibrillation attempts. By comparison, the cumulative defibrillation energy was significantly lower in sildenafil group than in sildenafil + L-NAME group and SA group (p< 0.05). ROSC was achieved in eight piglets in the both sildenafil group and in the sildenafil + L-NAME group, and in seven of eight piglets in the SA group. In the SA group, six piglets died at 14mins, 70 mins, 196 mins, 256 mins, 322 mins and 416 mins after ROSC. In the sildenafil + L-NAME group, four piglets died at 10 mins, 75 mins, 180 mins and 210 mins after ROSC. While, in the sildenafil group, only one piglet died at 48 minute after ROSC. |
| - **Adverse events** | **17** | **Yes** |
| **DISCUSSION** |  |  |
| - **Interpretation/scientific implications** | **18** | **Yes** |
| - **Generalisability/translation** | **19** | **Yes** |
| - **Funding** | **20** | This work was supported by the National Natural Science Foundation of China (No. 81601660) (to Z Q). The funder had no role in study design, data collection and analysis, decision to publish, or preparation of the manuscript. |
